# Marine Biological Macromolecules and Chemically Modified Macromolecules; Potential Anticoagulants

**DOI:** 10.3390/md20100654

**Published:** 2022-10-21

**Authors:** Pathum Chandika, Pipuni Tennakoon, Tae-Hee Kim, Se-Chang Kim, Jae-Young Je, Jae-Il Kim, Bonggi Lee, BoMi Ryu, Hyun Wook Kang, Hyun-Woo Kim, Young-Mog Kim, Chang Su Kim, Il-Whan Choi, Won Sun Park, Myunggi Yi, Won-Kyo Jung

**Affiliations:** 1 Marine Integrated Biomedical Technology Center, The National Key Research Institutes in Universities, Pukyong National University, Busan 48513, Korea; 2 Research Center for Marine Integrated Bionics Technology, Pukyong National University, Busan 48513, Korea; 3 Major of Biomedical Engineering, Division of Smart Healthcare and New-Senior Healthcare Innovation Center (BK21 Plus), Pukyong National University, Busan 48513, Korea; 4 Major of Human Bioconvergence, Division of Smart Healthcare, Pukyong National University, Busan 48513, Korea; 5 Major of Food Science and Nutrition, Pukyong National University, Busan 48513, Korea; 6 Department of Marine Biology, Pukyong National University, Busan 48513, Korea; 7 Major of Food Science and Technology, Pukyong National University, Busan 48513, Korea; 8 Department of Orthopedic Surgery, Kosin University Gospel Hospital, Busan 49267, Korea; 9 Department of Microbiology, College of Medicine, Inje University, Busan 47392, Korea; 10 Department of Physiology, Institute of Medical Sciences, School of Medicine, Kangwon National University, Chuncheon 24341, Korea

**Keywords:** blood coagulation, anticoagulant, marine sources, marine anticoagulant macromolecules, chemically modified macromolecules

## Abstract

Coagulation is a potential defense mechanism that involves activating a series of zymogens to convert soluble fibrinogen to insoluble fibrin clots to prevent bleeding and hemorrhagic complications. To prevent the extra formation and diffusion of clots, the counterbalance inhibitory mechanism is activated at levels of the coagulation pathway. Contrariwise, this system can evade normal control due to either inherited or acquired defects or aging which leads to unusual clots formation. The abnormal formations and deposition of excess fibrin trigger serious arterial and cardiovascular diseases. Although heparin and heparin-based anticoagulants are a widely prescribed class of anticoagulants, the clinical use of heparin has limitations due to the unpredictable anticoagulation, risk of bleeding, and other complications. Hence, significant interest has been established over the years to investigate alternative therapeutic anticoagulants from natural sources, especially from marine sources with good safety and potency due to their unique chemical structure and biological activity. This review summarizes the coagulation cascade and potential macromolecular anticoagulants derived from marine flora and fauna.

## 1. Introduction

Localized blood clot formation, and thereby hindering the blood flow through the circulatory system, is a foremost cause of morbidity and mortality in high-income countries, and the incidence is dramatically increasing in the rest of the world; associated with arterial diseases such as ischemic stroke, unstable angina, vein thrombosis including deep vein thrombosis (DVT) and pulmonary embolism (PE), and myocardial infarction (MI) [1,2]. Various antithrombotic treatments including antiplatelet drugs, fibrinolytic agents, and anticoagulants (blood thinners) are currently used for acute treatment and prevention of thrombosis [3]. Anticoagulant agents are chemically heterogeneous groups of drugs that target different factors of the coagulation cascade and are parenteral and oral agents which are typically used for initial treatment and long-term management of both arterial and venous thrombotic disorders [4,5].

Heparin and heparin-based anticoagulant and vitamin K antagonists are the main two classes of anticoagulants that have dominated in prescribed classes of anticoagulant medication for over half a century [6]. Heparin and heparin-based anticoagulants act as indirect anticoagulants that bind to the innate anticoagulant (antithrombin) and interact with various clotting factors of the coagulation cascade and enhance the anticoagulation capacity. Heparin and heparin-based anticoagulants are injectable anticoagulants prescribed for short-term prophylaxis of thrombosis, and with their prescription limited to an in-patient setting or venous thromboembolic (VTE) prophylaxis. In addition, unfractionated heparin requires additional monitoring and dose adjustment due to unpredictable pharmacokinetic and pharmacodynamic properties and non-hemorrhagic side effects such as heparin-induced thrombocytopenia (HIT) and osteoporosis, which develop via heparin binding to the cell and plasma proteins apart from antithrombin [7,8]. Warfarin is a vitamin K antagonist which activates several factors in the coagulation cascade and it was the most commonly prescribed and orally bioavailable long-term anticoagulant therapy [9] prior to direct oral anticoagulants (DOACs) such as dabigatran, followed by rivaroxaban, apixaban, edoxaban, and betrixaban, in the following years being approved by the US Food and Drug Administration, since warfarin has a risk of bleeding, unpredictable pharmacokinetic, drug interaction, and difficulty in dose adjustments [10]. However, the shortcomings of existing anticoagulants and ever-increasing global market opportunities (https://www.researchandmarkets.com/reports/5317270; accessed on 16 July 2022) resulted in continuous interest in the search for new or alternative anticoagulants from a natural resource that would be most effective as prevailing or with enhanced efficiency and safety.

Marine organisms represent a wide reservoir of remarkable bioactive molecules with an extraordinary diversity of functional and structural properties. Bioactive molecules extracted from marine flora, fauna, and microorganism, display a full spectrum of properties and are utilized in multiple applications. From bioactive secondary metabolites to biological macromolecules such as polysaccharides and proteins extracted from marine natural sources, they have been increasingly employed in medicinal and pharmaceutical applications such as cancer and chronic diseases treatments, and tissue engineering applications, cell therapies, and the cosmetic industry [11,12,13,14]. Importantly, due to the broad structural and functional diversity, insignificant toxicity, great variety of mechanisms of action, promising biological properties, traditional medicinal value, and high potential for subjecting those into various chemical modifications, marine-derived biological macromolecules have been well studied and documented as promising and potential alternative anticoagulants. In this review, we summarize the research on marine-derived substances that have a positive effect on prolongation of blood coagulation via interaction with inborn anticoagulants and clotting factors in the coagulation cascade.

## 2. Coagulation

**Figure 1:**
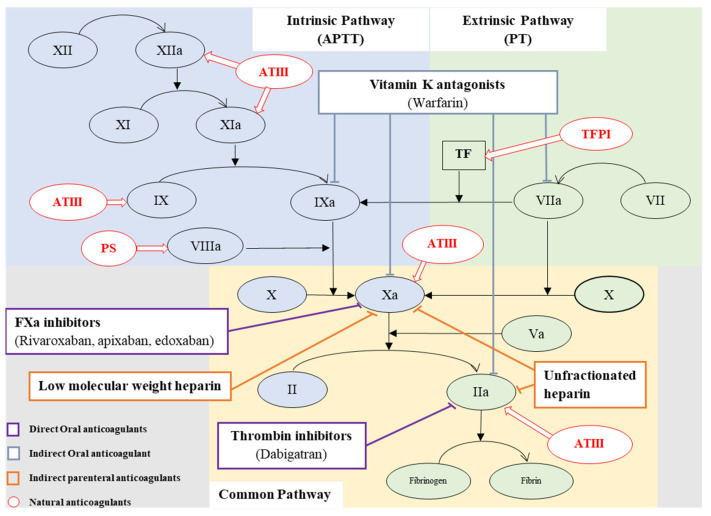
Coagulation cascade, natural and current anticoagulants, and their targets in the coagulation cascade.

Blood coagulation is a process that activates immediately with the rupture of endothelium followed by exposure of the tissue factor and collagen to circulating blood, which converts liquid blood to an insoluble gel involving platelet activation, adhesion, aggregation, fibrin deposition, and maturation [15]. Coagulation is initiated with two main pathways, intrinsic and extrinsic, followed with the common pathway (Figure 1), where the intrinsic pathway is dependent on contact activation by a negatively charged surface and involves factor XII, XI, IX, and VIII, and the extrinsic pathway requires a tissue factor which is extrinsic, not normally circulating in blood vessels, and also factor VII [16]. Both intrinsic and extrinsic pathways converge on the common pathway to activate factor X, leading to conversion of prothrombin to thrombin and ending the conversion of fibrinogen to fibrin, the arterial thrombus. Except for factor III, IV, and VIII, most of the factors involved in the coagulation cascade are produced by the liver [17].

The extrinsic pathway gets activated when the vessel wall disrupts and exposes the tissue factor (TF) to the circulating plasma factor VII (FVII) or activated factor VII (FVIIa), and it makes the TF-FVIIa complex and activates both factor IX (FIX) and factor X (FX). At the propagation phase, factor Va (FVa) combines with factor Xa (FXa) and a portion of it catalyzes the conversion of prothrombin to thrombin, and it induces the local platelet aggregation and activation of cofactors V and VIII; the other part of Xa functions as negative feedbacks of the extrinsic pathway by forming complexes with tissue factor pathway inhibitors one and two (TFPI-1 and TFPI-2). In the intrinsic pathway, thrombin is formed through FIXa with its cofactor VIIIa and thrombin hydrolyses the bonds of fibrinogen to form fibrin monomers, which simultaneously activates FXIII, which cross-links the fibrin and improves tensile strength. The formation of the fibrin strands represents the second phase of hemostasis [18,19].

## 3. Natural Anticoagulants

Natural anticoagulants are produced outside the coagulation pathway by the body itself, and act as dampers avoiding excessive coagulation and formation of blood clots which obstruct normal blood flow. A deficiency of one of those natural anticoagulants may imbalance the clotting process and lead to thrombophilia, too much clotting. Thrombus formation is inhibited by the main natural anticoagulants including antithrombin III, heparin cofactor II, protein C, protein S, and TF inhibitor [20]. Antithrombin, the main inhibitor of thrombin, is a serine protease inhibitor; it binds and inactivates thrombin, FIXa, FXa, FXIa, and FXIIa in the presence of heparin and heparin cofactor II (HCII) [18,21,22]. The tissue factor plasminogen inhibitor is a polypeptide produced by endothelial cells which play as a natural inhibitor of the extrinsic pathway by inhibiting the TF–VIIa complex. The protein C pathway, which includes Protein C, Thrombomodulin, Endothelial protein C receptor, and Protein S, inhibits the propagation phase of coagulation. Protein C is a serine protease activated by thrombin and it inhibits FVa and FVIIIa with cofactors Protein S and phospholipids; thrombomodulin, a transmembrane receptor, binds to thrombin and prevents the formation of clots; and Endothelial protein C receptor helps in the activation of Protein C. Protein S is a vitamin K-dependent glycoprotein, and it functions as a cofactor to activated protein C (APC) in the inactivation of FVa and FVIIIa and causes direct reversible inhibition of the prothrombinase (FVa–FXa) complex [23].

## 4. Thrombosis

A balance between clotting and bleeding is always maintained in the body, though any pathological scenario will change this balance to either hemorrhagic or thrombotic complications. Thrombosis is the imbalanced state of intravascular activation of coagulation, due to deficiencies in natural anticoagulants, forming a blood clot in the blood vessels; if the clot dislodges and gets blocked in a vein, venous thrombosis occurs, and if in an artery, arterial thrombosis occurs (atherothrombosis). Thrombosis may lead to serious consequences of MI, stroke, PE, DVT, and disseminated intravascular coagulation (DIC) and it would cause a higher amount of morbidity and mortality every year worldwide [24]. An arterial thrombus is rich in platelets and poor in fibrin, because it forms under conditions of higher pressure and shear forces, while a venous thrombus is primarily composed of fibrin and red blood cells, giving it the appearance of red clots. When a venous blood clot dislodges and moves to the lungs, it gives rise to PE, and venous thromboembolism occurs when PE comes along with venous thrombosis [25]. Platelet activation is more important in the pathogenesis of arterial thrombosis, while activation of blood coagulation is more critical and platelet activation is less important in pathogenesis of VTE. Anticoagulants and drugs which suppress platelet function are more effective for the treatment of arterial thrombosis and anticoagulant therapy is more effective for prevention and treatment of VTE, but prevention through lifestyle interventions for smoking, obesity, and inappropriate dietary habits should be the community goal.

## 5. Anticoagulant Therapy

Anticoagulant therapy (Figure 1) reduces the activity of proteases or cofactors, targeting the action of thrombin or its generation by imitating the function of natural anticoagulants on its deficiency. Several types of anticoagulants are presently identified and practiced and those act on several places of the coagulation cascade to prevent thrombosis by inhibiting the coagulation mechanism. Current anticoagulant therapy can be categorized into indirect thrombin inhibitors, direct thrombin inhibitors, direct FX inhibitors, and vitamin K antagonists, or else as heparin, warfarin, and direct oral anticoagulants (DOACs). Heparin is administered parenterally, while warfarin, dabigatran, and rivaroxaban are orally administered drugs [26,27,28,29,30]. Novel anticoagulants are now in therapeutic use, which target specific coagulation factors such as FXa and thrombin [19]. Vitamin K antagonists such as warfarin were the only available oral anticoagulant since the 1950s, considered as the gold standard and as commonly prescribed for long-term use. Warfarin prevents coagulation by inhibiting the C1 subunit of the vitamin K epoxide reductase enzyme, consequently down-regulating the synthesis of the clotting factor and prothrombin, FX, FVII, FIX, protein C, and protein S. Warfarin is restricted to a narrow therapeutic window and requires periodic dose adjustments and laboratory monitoring to ensure that the patient remains within the target International Normalized Ratio (INR), which balances the hypercoagulability and risk of bleeding complications [31]. Animal-derived unfractionated heparin (UFH) and chemically fractionated low molecular weight heparin (LMWH) are indirect thrombin inhibitors and rapid anticoagulants, which bind to antithrombin and inhibit FXa. UFH has a short half-life and therapeutic efficacy occurs immediately, whereas warfarin takes a couple of days to fully manifest the anticoagulation effect. UFH inhibits thrombin, FXa, FVIIa-TF complex, FIXa, FXIa, and FXIIa. LMWH has a half-life of 3–4 h, with more predictable action, less regular laboratory monitoring, fewer bleeding complications, and is stable for long-term anticoagulation [32]. The limitations of currently available anticoagulant treatments have prompted the development of novel anticoagulants over decades, administered orally or parenterally, which do not require routine coagulation monitoring. DOACs, such as Rivaroxaban, Apixaban, and Edoxaban, inhibit FXa, and Dabigatran inhibits thrombin directly; they have shown their ability and safety for prolonged complicated treatments [33].

## 6. Issues Related with Current Anticoagulants

Vitamin K antagonists like warfarin, administered orally, are challenging due to long plasma half-life, slowness in action, and the need for regular laboratory checkups due to increased bleeding tendency and their ability to interact with food, drugs, and genetic polymorphisms. Heparins like UFH and LMWH are administered parenterally and need routine dose adjustments and regular anticoagulant monitoring due to the high risk of bleeding associated with the inability to neutralize fibrin-bound thrombin and less rapid reversal action in the occurrence of overdose [4]. Though UFH is rapid, the dose-dependent anticoagulant response is unpredictable because heparin binds non-specifically to various plasma proteins. Obsessed patients have complications because current anticoagulant dosing is based on total body weight, rather than considering the ideal body weight. Heparins and vitamin K antagonists have antidotes which reverse their therapeutic effect [34]. Though the novel oral anticoagulant has already shown its efficacy compared with the previous anticoagulant, it is also accompanied by some limitations. Still, DOACs are used in clinical trials and approved for usage in limited countries, and can be used only for patients whose indications were studied previously in clinical trials, though many not studied indications may be remaining without eligibility for treatment [35]. Routine monitoring is required in circumstances like overdose, but previously routinely used INR cannot be applied for some treatments like rivaroxaban because DOACs have different targets and variable effects on routine coagulation assays. DOACs have a shorter half-life compared to warfarin and the requirement for frequent dosing may result in the rapid abolition of anticoagulant protection since the missing of any dose will critically affect the patient. [36]. DOACs are more expensive compared to traditional anticoagulant therapies which also need to be considered to optimize the outcomes.

## 7. Marine-Derived Anticoagulant

Marine organisms, which make up of nearly one half of total global diversity, are a rich source of structurally diverse bioactive and biocompatible materials with numerous biological activities. This is because marine organisms live in a very competitive and exigent environment, a state that demands the production of specific molecules [37]. With the difficulties in exploring the entire ocean habitat, various materials have yet to be isolated and identified; thus, the importance of the marine organism as a reservoir of various biologically active materials has continued to grow over the decades [38,39,40]. Therefore, continuous attention has been paid over the decades to unraveling structural and compositional properties of various marine-derived materials and identification of their potentials in anticoagulation activity.

## 8. Sulfated Polysaccharides

### 8.1. Glycosaminoglycans

**Figure 2:**
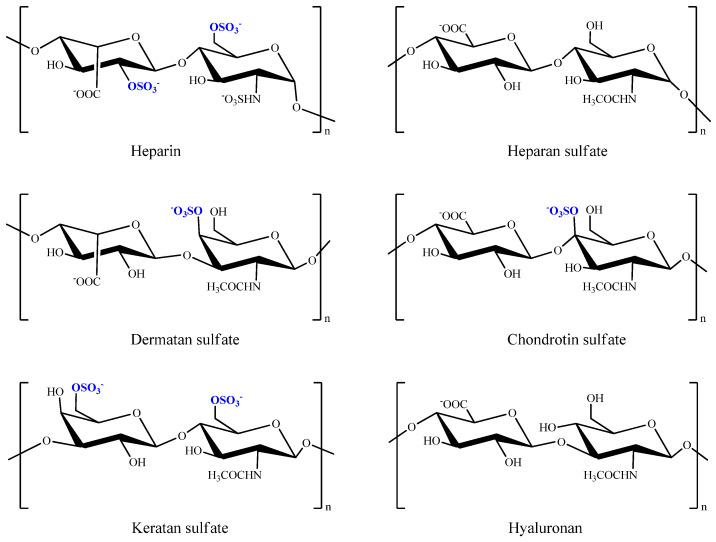
Chemical structures of sulfated and non-sulfated glycosaminoglycans fragments.

Glycosaminoglycans are sulfated anionic polysaccharides composed of repeated *O*-linked disaccharide of hexosamine (glucosamine and galactosamine) and uronic acid (glucuric acid and iduronic acid) or galactose, extensively found in the extracellular matrix and on the cell surface of the animal tissue. Glycosaminoglycans derived from marine algae and animals such as heparin, heparin sulfate, dermatan sulfate, fucosylated chondroitin sulfate, chondroitin sulfate, keratan sulfate, (Figure 2) and glycosaminoglycans mimetics differ from those present in terrestrial organisms in both sulfate characters and molecular weight [41,42,43], even though most of those glycosaminoglycans are reported to have anticoagulant properties depending upon their structural composition and arrangements, sulfate content and substitution pattern; keratan sulfate and hyaluronan were not reported to exhibit anticoagulant properties. However, only a sulfated hyaluronate has been reported to have anticoagulant properties [44,45,46], but there was no strong evidence in the recent past to support those findings.

#### 8.1.1. Heparin and Heparin Sulfate

Heparin was originally introduced to prevent thrombosis in surgical patients, followed by use in treating deep venous thrombosis and in preventing various complications following vascular surgery and MI [47]. Heparin is primarily extracted from porcine and bovine and has been widely used since 1930 as an anticoagulant drug, but safety issues and increasing demand have encouraged a search for alternatives [48]. Marine animal-derived heparin and heparin sulfate could be an alternative since they are widely studied and some of them are structurally similar to the mammalian heparin. Heparin and heparin sulfate are structurally similar in their polysaccharide chains and are composed of 1 → 4 linked disaccharide units, comprising β-d-glucuronic acid or α-l-iduronic acid and α-d-glucosamine with variable modification patterns occurring at several positions [49]. Importantly, an alternative marine-derived heparin should have to be safe and should have anticoagulant properties comparable to mammalians such as heparin from bovine intestinal mucosa (150 USP units/mg) [50] and porcine mucosa (180 USP units/mg) [51] and is extractable in adequate quantities. Setting up porcine mucosa as a benchmark, the heparin sulfate isolated from mollusk *Nodipecten nodosus* showed 5-fold low anticoagulant activity (36 USP units/mg) as measured by the APTT assay. In addition, that could inhibit in vitro FXa (IC50 0.835 g/mL) and thrombin (IC50 9.3 g mL/L) in the presence of antithrombin and in vivo formation of thrombus in photochemically injured arteries [51]. In the case of heparin sulfate isolated from different mollusks species *Tridacna maxima* and *Perna viridis*, it has demonstrated low anticoagulant activity, 7.4 USP units/mg and 4.3 USP units/mg, respectively, but with higher yields of 20,128 USP units/kg and 9460 USP units/kg respectively [52]. However, the clam *Tapes philippinarum* had not only a higher heparin yield and anticoagulant activity, but had identical antithrombin III (ATIII) binding sites, similar to that of human, porcine, and bovine intestinal mucosal heparins [53]. In addition to heparin isolated from mollusks, some other invertebrates-derived heparin and heparin sulfates have also demonstrated considerable anticoagulant potential exhibited by prolongation of coagulation pathways (Table 1).

#### 8.1.2. Chondroitin/Dermatan Sulfate

Chondroitin sulfate is a linear polysaccharide composed of a repeated disaccharide unit containing *N*-acetyl-β-d-galactosamine and β-d-glucuronic acid, which was sulfated in the carbon 6, 4 at *N*-acetyl galactosamine, both 4 and 6, and positions 6 of GalNAc and 2 of d-glucuronic acid [54]. Disaccharide units containing a hexosamine, *N*-acetyl galactosamine, and l-iduronic acid joined by β 1,4 or 1,3 linkages, respectively, and commonly sulfated at position 4 of *N*-acetyl galactosamine, are dermatan sulfate [55]. Dermatan sulfate was isolated from three species of rays from the Brazilian seacoast, *Dasyatis americana*, *Dasyatis gutatta, Aetobatus narinari*, and freshwater *Potamotrygon motoro* composed of mono-sulfated disaccharides, and disulfated disaccharides bearing esterified sulfate groups at different positions (C-n) was shown with different anticoagulant activities depending on the composition and arrangements of the disulfated disaccharide. *D. gutatta* had shown similar anticoagulant activity to that of the mammalian, whereas *D*. *Americana* had higher APTT and HCII-mediated inhibition of thrombin [56]. In addition to dermatan sulfate and chondroitin sulfate alone demonstrating various extend of anticoagulant activities (Table 1), a mixture of both chondroitin and dermatan sulfates at specific ratios have also been reported. The mixture of chondroitin and dermatan sulfates isolated from both the skin and bones of corb (*Sciaena umbra*) were evaluated in vitro using APTT, TT, and PT, and demonstrated that both skin and bone extract could prolong the APTT by 1.59 and 1.48-fold, respectively [57]. More recently, it was validated that a mixture of chondroitin and dermatan sulfate isolated from the skin of corb (*Sciaena umbra*) was in a 1 to 3 ratio, and that it has a remarkably high anticoagulant effect, while with 1000 µg/mL of concentration, it could significantly prolong the clotting time by 2.48-fold [58].

#### 8.1.3. Fucosylated Chondroitin Sulfate

Fucosylated chondroitin sulfate is sea cucumber-derived, an uncommon sulfated glycosaminoglycan composed of a chondroitin sulfate like backbone consisting of alternating β-1,4-linked D-glucuronic acid and β-1,3-linked *N*-acetyl-d-galactosamine disaccharide units with α-L-fucose branches linked to the *O*-3 position of β-1,4-linked D-glucuronic acid residues [74], and they are markedly different to typical mammalian glycosaminoglycan due to uniqueness in sulfated fucose side chains [75]. Recently, relatively low molecular weight (36.3 kDa) fucosylated chondroitin sulfate was isolated from *Cucumaria syracusana* (with ∼35.6 mg/g dry body wall) consisting of chondroitin sulfate backbone branched by two types of fucose 2,4-*O*-di and 3,4-*O*-disulfated residues in respective ratios of 57.5 and 42.5 %. The anticoagulant activity revealed that they have high anticoagulant properties mediated by the HCII and slightly by the antithrombin with IC_50_ 0.05 µg/mL and 0.09 µg/mL, respectively [70]. Several more fucosylated chondroitin sulfates isolated from sea cucumber species have been demonstrated to have high potential anticoagulant activity (Table 1). Even though most of their anticoagulant activity exhibited is related to the HCII-dependent thrombin inhibition, and ATIII mediation [70,73,76], the most prominent anticoagulant mechanism could be the inhibition of the FXa production by the intrinsic tenase complex [66,72,73]. Though these sea cucumber-derived fucosylated chondroitin sulfates show significant anticoagulant activity, they also show some undesired effect such as platelet aggregation and FXII activation [77,78]. Therefore, β-eliminative depolymerization has been performed with *Thelenota ananas*-derived fucosylated chondroitin sulfate by treating benzyl esters with alkaline to cleave the glycosidic linkages of GalNAc-β1, 4-D-glucuronic acid. The resultant fragments demonstrated potential anticoagulant activity by inhibiting the intrinsic tenase while diminishing or eliminating the activation of FXII and the platelet [71].

### 8.2. Glycosaminoglycans Mimicking

#### 8.2.1. Ulvan

Ulvans are water soluble sulfated polysaccharides extracted from green algae, and are arranged in diads consist of sulfate, rhamnose, uronic acids, and xylose residues to form ulvanobiuronic acid type A (A_3s_): (→4)-α-l-Rha*p* 3-sulfate-(1→4)-β-d-GlcA*p*-(1→), type B (B_3s_): (→4)-α-l-Rha*p* 3-sulfate-(1→4)-α-l-IdoA*p*-(1→), ulvanobiose 2′,3-disulfate (U_2′s,3s_): (→4)-α-l-Rha*p* 3-sulfate-(1→4)-β-d-Xyl*p* 2-sulfate-(1→), and ulvanobiose (U_3s_): (→4)-α-l-Rha*p* 3-sulfate-(1→4)-β-d-Xyl*p*-(1→) [79]. In recent years, ulvan has proven to have remarkable biological properties such as antiviral and antitumoral and also as a biomaterial in tissue engineering and drug delivery applications. However only a few studies have been reported for its anticoagulant activity. The relationship between the ulvan chemical structure and its anticoagulant properties is complex, yet sulfate content and substitution pattern may significantly govern the anticoagulant activity [80]. More recently, ulvan (1→4)-β-glucuronic acid, (1→3,4)-α-L-rhamnose-3-sulphate and (1→4)-α-xylose) isolated from *Ulva lactuca* prolonged the APTT and TT and also moderated PT clotting time suggesting that extracted ulvan inhibits the intrinsic blood coagulation pathways and/or thrombin activity [81]. In another study, ulvan isolated from *U. lactuca* exhibited excellent anticoagulant activity which was analyzed using in vitro APTT, PT, TT, factor Xa, and IIa. The in vivo antithrombotic activity analyzed by the vanae cavae ligature experimental rat model showed that ulvan reduced the weight of thrombus which was associated with the Fxa and FIIa of the common pathway intermediated by ATIII [82]. Moreover, ulvan isolated from various other green algae such as *Ulva conglobate*, *Ulva reticulata*, *U. fasciata*, and *Ulothrix flacca* (Table 2) provides strong evidence about the anticoagulant potential of ulvans isolated from green algae. The potential anticoagulant activity of ulvan also suggested to enhance by chemically doubling the sulfate content of ulvan. In this study, ulvan extracted from *U. rigida* was chemically sulfated using the sulfur-trioxide pyridine complex (SO_3_–pyridine) method, and in dimethylformamide (DMF) and pyridine, had shown a stronger anticoagulant property which was investigated through the intrinsic, extrinsic, common, and specific antithrombin-dependent pathway; stronger than some of the commercial anticoagulants such as heparin and Levonox, indicating that chemically sulfated ulvan could be a high potent alternative [80]. The effect of the low molecular weight ulvan isolated from marine green algae *U. flacca* with alternate 4-linked-α-L-rhamnose residues and 4-linked-β-D-glucuronic acid residues on both intrinsic and common coagulation pathways showed a prolonged clotting time, 200 s for APTT and 120 s for TT for the 20 μg/mL ulvan concentration [83].

#### 8.2.2. Carrageenan

**Figure 3:**
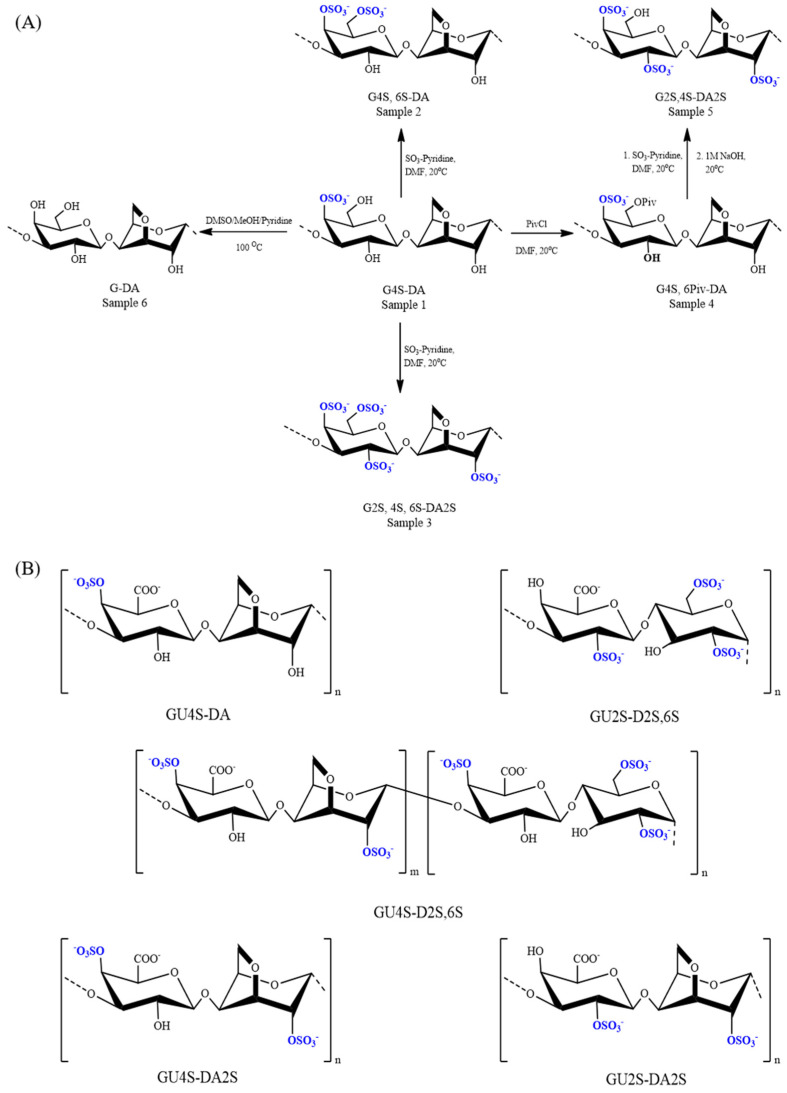
Structures of kappa carrageenan. (**A**) Selective sulfation and desulfation of kappa carrageenan. (**B**) Structures of C-6 oxidized carrageenan [89,90].

Carrageenans are a complex of sulfated galactan obtained from red algae which are composed of repeating disaccharide units of (1→3)-linked β-d-galactopyranose and (1→4)-linked α-d-galactopyranose, in which the α unit can be found as the 3,6-anhydro derived. In addition sulfate groups are bound to specific hydroxyl groups forming several sulfation possibilities in the carrageenan polysaccharide backbone [107]. At least 17 different types of carrageenan have been identified and some have been recognized for a broad spectrum of biological activities including anticoagulant activity (Table 2), which are dependent upon the sulfation pattern [88,108]. In the comparisons of the commercially most available carrageenans types such as kappa, iota, and lambda carrageenans, it was reported that lambda carrageenan has higher anticoagulant potential through both intrinsic and extrinsic pathways [88] and especially, high molecular weight lambda carrageenan can be comparable to the anticoagulant activity of commercial anticoagulants [86] because of the amount and the position of the sulfate groups. The sulfate regiochemistry has demonstrated that the synthesis of selective chemically sulfated carrageenan (Figure 3), which were sulfated at C6 of β-d-Gal*p* and C2 of 3,6-anhydro-α-d-Gal*p* units, could enhance the anticoagulant activity of carrageenan [89]. In addition, several researchers have shown that the anticoagulant activity of carrageenan can be enhanced by the oxidization of C6 of β-d-Gal*p* units using 2,2,6,6-tetramethylpiperidine-1-oxyl (TEMPO) and trichloroisocyanuric acid (TCCA) in bicarbonate buffer (Figure 3). This enhancement of the anticoagulant property was explained due to the synergetic property of carboxylic groups bound after the oxidization and the native sulfate groups [90]. Moreover, carrageenan has become a promising biomaterial to provide anticoagulant properties to various applications such as the fabrication of gel beads [93], and composite hydrogels [92].

#### 8.2.3. Fucoidan or Fucan Sulfate

Fucoidan or fucan sulfate is a sulfated polysaccharide obtained mainly from marine brown algae or some invertebrates such as the sea urchin and sea cucumbers. Fucoidans, found mainly in brown algae, are branched sulfated fucan and complex heteropolysaccharide, consisting of the linear backbone with (1 → 3)-α-L-Fuc or alternating (1 → 3)-α-L-Fuc and (1 → 4)-α-L-Fuc units with sulfate groups often found at the *O*-2, and/or *O*-3 and *O*-4 positions. Sulfated fucan, found in the sea cucumber, is structurally simpler compared with fucoidan and consists of the linear backbone with (1 → 3)-α-L-Fuc, or alternating (1 → 3)-α-L-Fuc and (1 → 2)-α-L-Fuc units with sulfate groups frequently found at the *O*-2 and/or *O*-4 positions of fucose [99,109]. Fucoidan has been reported to possess various biological activities such as anticancer, immunomodulating, antiviral, antiangiogenic, antioxidant, and antitumor activities. By far, the anticoagulant activity of these sulfated polysaccharides is the most widely studied, due to the high intention in searching for substitutes for heparin [109,110,111] (Table 2). Weihua and his coworker evaluated eleven fucoidans; seven that differed with respect to the average molecular weight and another four with respect to both the molecular weight and molar ratio of fucose and galactose. This study clearly demonstrated that fucoidans exhibit appropriate anticoagulant activity; that demonstrated that not only the average molecular weight but also the fucose and galactose ratio of fucoidans play an important role in anticoagulation [94]. In addition, low molecular weight fucoidan obtained from brown seaweed *Laminaria japonica* separated into three fractions also showed suitable anticoagulant activity. This study also showed that the molar ratio of sulfate/fucose and sulfate/total sugar, sulfate group content, and the molecular weight of fucoidan play an important role in the anticoagulant activity [97]. The high molecular weight fucoidan extracted from *Fucus vesiculosus* has been reported to significantly increase the prothrombin time when the concentration of fucoidan increased above 80 μg/mL, indicating that a high molecular weight of fucoidan has a greater effect towards the greater anticoagulation [112]. Controversy, depolymerized fractions of fucoidan from *F. vesiculosus* showed negligible anticoagulant activity and FXII activating potency indicating that low molecular weight factions do not affect blood coagulation [113]. However, highly sulfated chemically transformed branched xylofucan has been shown to effectively increase inhibition of clot formation compared to pure material. The experiment with purified fucoidan derivatives with an average 2.0 degrees of sulfation could exhibit antithrombin-mediated thrombin inhibition similar to that of Clexane [96]. In addition, different extraction techniques such as ultrasound-assisted extraction promote the total fucoidan extract from the alga samples [114] and this has been found to affect the availability of sugar content and the sulfate groups, which is directly proportional to the ability of fucoidan to enhance the anticoagulant activity [115]. Sulfated fucans extracted from invertebrates have also been investigated and the results clearly demonstrated their high anticoagulant potential *(*Table 2).

#### 8.2.4. Rhaman Sulfate

The availability of sulfated polysaccharides that are mainly composed of α-l-rhamnose is limited in the marine source and so far, limited reports are available on its structural and biological properties. *Monotroma* is a genus of marine green algae that present sulfated rhaman, and it is reported to exhibit anticoagulant, anticancer, antiviral, and immunomodulatory activities. However, attention on the anticoagulant activity of sulfate rhamans derived from the genus *Monotroma* is particularly augmented [116] (Table 2). Hongyan and his research team reported anticoagulant rhaman sulfate from *Monostroma* *latissimum* composed of (1 → 3)-linked α-l-rhamnopyranose, (1 → 2)-linked α-l-rhamnopyranose, and (1 → 2,3)-linked α-l-rhamnopyranose residues in a molar ratio of 4:1:1, with the sulfate groups at C-2 and C-3 α-l-rhamnopyranose residues; which was found to effectively increase the clotting time in the APTT and TT assays proportional to the concentration. A different rhaman-type sulfated polysaccharide from *Monostroma angicava* which consists of → 3)-α-l-*R*ha*p*-(1→ and →2)-α-l-*R*ha*p*-(1 → residues, branches at C-2 of → 3)-α-l-*R*ha*p*-(1 → residues, with sulfate groups at C-3 of  → 2)-α-l-*R*ha*p*-(1 →  residues was found to possess high anticoagulant activity, mainly attributed to strong potential thrombin by HCII [102]. In addition to those, several more studies on those species and *Monostroma nitidum* have reported greater anticoagulant properties (Table 2).

### 8.3. Chemically Sulfated Polysaccharides and Oligosaccharides

#### 8.3.1. Sulfonated and Sulfated Chitosan and Chitosan Derivatives

**Figure 4:**
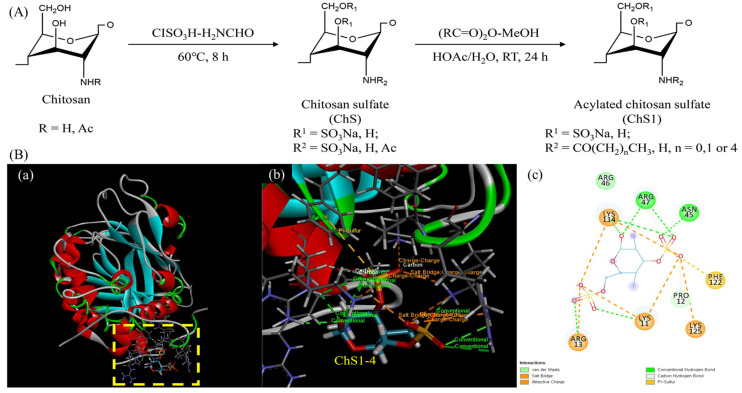
(**A**) Synthesis of acylated chitosan sulfate and its in silico analysis with antithrombin III. (**B**) (**a**) Hypothetical model of antithrombin III with ChS1-4. (**b**) 3D structure of ChS1-4. (**c**) 2D view of ChS1-4 and its interaction with antithrombin III [127].

Chitosan is a 1,4-β-linked copolymer composed of two repeat d-glucosamine and *N*-acetyl-d-glucosamine units, and which is obtained by the full or partial deacetylation of chitin, a naturally abundant cationic mucopolysaccharide that can be isolated from the crustacean shells and the cell walls of some fungi [117]. Chitosan is a naturally cationic polysaccharide that has a large number of free amino groups, and those functional groups provide chitosan to process attractive physicochemical and biological characteristics, allowing it to be suggested for various biomedical and pharmaceutical applications [118]. However, various researchers have turned to chemical modifications such as carboxyalkylation [119], hydroxyalkylation [120], and quaternization [121] to enhance the characteristic properties. The chemical changes at chitosan are supposed to occur generally in nucleophilic amino groups at the C-2 position or in the hydroxyl groups at both acetyl glucosamine and glucosamine repeat units at C-3 and C-6 positions, or maybe in hydroxyl and amino groups. Especially, chemical sulfonation of chitosan has gained significant attention from researchers on various modifications. The sulfonate groups can be directly coupled with amino groups leading to production of sulfamate products (−NH-SO_3_^−^), or by sulfonate groups (R-SO_3_^−^) leading to sulfonated products (−NH-R-SO_3_^−^). In addition, this can occur in the hydroxyl groups, leading to the production of sulfated products (−O-SO_3_^−^)[122,123]. This modified chitosan processes residual amino groups and attaches sulfonate or sulfated products, which provide sulfated chitosan to similar characteristics to that of sulfated glycosaminoglycans. Therefore, most researchers have focused on investigating anticoagulant properties of sulfated chitosan and chitosan derivatives. Several researchers have recently synthesized sulfonated chitosan by the reaction of chitosan with chlorosulfonic acid in *N*,*N*-dimethylformamide and demonstrated excellent anticoagulant properties through PT and APTT [124,125] and through the in vivo tail bleeding method in the Wister rat. The developed chitosan sulfate demonstrated faster onset action compared to the standard (nicoumalone) after one hour of the administration [126]. Our previous results also demonstrated excellent anticoagulant activity through both intrinsic and common pathways, mediated through ATIII, mainly involving FXa and FIIa. Molecular docking also validates the interaction of sulfated chitosan and ATIII (Figure 4) [127]. Similar to our study, sulfonated low molecular weight chitosan also showed the ATIII-mediated anticoagulant property [128]. In addition, various chemical-sulfonated and sulfated chitosan and low molecular weight chitosan (Table 3) including the introduction of the carboxyl group to chitosan sulfate by the acylation reaction [129], synthesizing N-succinyl chitosan using sulfating agent N(SO_3_Na)_3_ [130], and *N*-propanoyl-, *N*-hexanoyl-, and *N*,*O*-quaternary-substituted chitosan sulfate, showed promising anticoagulant activities [131]. Moreover, they have been incorporated for the fabrication of anticoagulant electrospun membranes for various further applications [132].

#### 8.3.2. Sulfated Alginate

**Figure 5:**
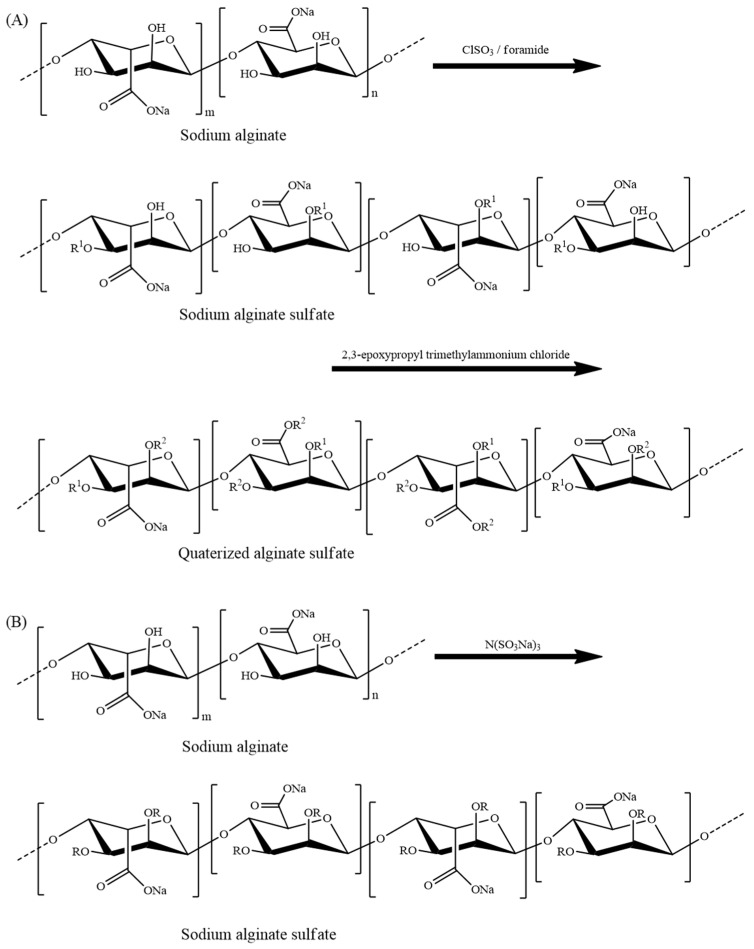
The synthesis of (**A**) sodium alginate sulfates and its (**B**) quaterized derivatives using different reagents. R = SO_3_Na or H, R^1^ = SO_3_Na, and R^2^ = CH_2_CH(OH)CH_2_N(CH_3_)_3_Cl [142,144].

Alginate is an anionic water-soluble, non-immunogenic, and biocompatible polysaccharide composed of 1→4 linked β-d-mannuronic acid (M) and its C-5 epimer α-l-guluronic acid (G), which has been widely used in diverse biomedical and pharmaceutical applications, especially in drug delivery and skin tissue regeneration applications [11,152]. However, the blood compatibility of alginate was questionable to meet the requirement in some cases. In order to match the blood compatibility, heparin was grafted onto alginate [153]. However, if the alginate was chemically sulfated, it could enhance the blood compatibility since sulfation would give alginate a structural similarity to that of heparin. Thus, various chemical strategies have been developed over the decades to sulfate the alginate (Figure 5A), aiming to enhance the anticoagulant properties (Table 3). Huang and his co-workers reported for the first time sulfation of alginate through the reaction with chlorosulfonic acid in formamide with a higher degree of sulfation. The anticoagulant activity was measured by the APTT, PT, and TT and resulted in greater anticoagulant activity than heparin. Since the higher anticoagulant activity gained due to the over-sulfation was not a merit in cases such as bleeding, quaternary ammonium groups were introduced into sulfated alginate to control the anticoagulant activity [144]. In addition, uncommon sulfating agent (N(SO_3_Na)_3_), which was synthesized by sodium bisulfite and sodium nitrite in an aqueous medium, was employed to synthesize alginate sulfate with a 1.87 degree of sulfation at optimum conditions by the Lihong and his co-workers. They also found that strong anticoagulant activity yet depended upon the degree of sufation and the molecular weight. They also found that a high concentration and degree of sulfation could inhibit the activity of FIIa and FXa to prolong the APTT and TT, while a low molecular weight alginate sulfate resulted in high anti-FXa [142]. Propylene glycol alginate sodium sulfate (PSS) is prepared from alginate via hydrolysis, esterification, and sulfation (Figure 5B), which is a drug with anticoagulant activity, hypotensive active, blood viscosity reductive functions, and which has been used in China for nearly four decades [147]. PSS is an excellent anticoagulant [146] (Table 3), but it was found that fractions of PSS, which has a low M/G unit ratio with higher molecular weight, lead to the excessive prolongation of APTT, TT, over inhibition of FIIa mediated by ATIII leading to the risk of bleeding [145]. Going beyond as an anticoagulant drug, various researchers focus on several fabrications such as the development of blood-contacting membranes, especially for blood purification issues. The research team has developed a sulfated alginate immobilized polyethersulfone hallow fiber membrane which enhances the required characteristics and could prolong the coagulation time (35s for APTT and 14s for PT) compared with a pristine polyethersulfone hallow fiber membrane, indicating enhancement of anticoagulant activity and indicating that sulfated alginate is a promising material for developing blood purification membranes [143].

### 8.4. Proteins and Peptides

Marine-derived bioactive and biocompatible proteins are often important in promoting health and reducing the risk of diseases. Hence, marine-derived bioactive proteins have been considered a part of functional food, medicine, and cosmetics [154]. Hence, we reported the first anticoagulant protein from marine bivalves (*Scapharca broughtonii*) with the prolongation of APTT and inhibition of FIX [155]. Then, in 2007, again we reported anticoagulant protein purified from the muscle protein of granulated ark (*Tegillarca granosa*, order Arcoida, marine bivalvia). The purified protein could satisfactorily prolong the TT, which corresponds to the FIIa inhibition. In addition, that inhibited the FVa and decreased the binding affinity of FVa to FII [156]. Similarly, enzymatically extracted marine fish, yellowfin sole (*Limanda aspera*) protein with anticoagulation activity initiated by inhibiting FXIIa, has also been reported by our research team [157].

Bioactive peptides are specific protein fragments that have been studied widely from various sources due to their numerous nutraceutical and medicinal values such as antimicrobial, antiviral, antioxidant, analgesic, anti-diabetic, neuroprotective, immunomodulatory, and anticoagulant activities. Due to the high potential, some marine derived peptides gained high commercial and market value and a considerably large number of marine peptides are at their clinical and preclinical level [158,159]. However, few studies of marine derive peptides have been reported with anticoagulant properties, which were evaluated using APTT, PT, and TT assays. Anticoagulant marine peptides were found in the algae *Porphyra yezoensis* (commercially known as Nori) [160], echiuroid worm (*Urechis unicinctus*) [161], goby muscle (*Zosterisessor ophiocephalus*) [162], bivalve mollusk (*Mytilus edulis*) [163,164], and oyster (*Crassostrea gigas*) [165], and those extracted marine peptides were found to prolong the coagulation with respect to the dose of the peptide. The peptide isolated from *U. unicinctus* with the 3344 Da molecular weight was reported to bound to FIXa and thereby inhibit the interaction between FIXa and FIX and prolong the clotting time (APTT from 32.3 ± 0.9 s to 192.8 ± 2.1 s) [161]. Similar to this study, our previous study that isolated peptide from *M. edulis* could inhibit the proteolytic activity of FX through the FXa and formation of FIIa through the prothrombinase complex and prolonged the clotting time by inhibiting the FX in the FIXa/VIIIa/PLs complex and the conversion of FII to FIIa in the FXa/FVa/PLs complex [164].

## 9. Clinical Use and Efficacy

Numerous investigations have been conducted over the past decades to isolate and identify marine-derived biological macromolecules as an alternative anticoagulant, and they have been confirmed with potential anticoagulant properties in vitro and in vivo settings; nevertheless, most of those are not yet evaluated for clinical use and efficacy. However, PSS was the first Chinese food and Drug Administration (CFDA)-approved heparinoid anticoagulant to be prescribed for DVT, MI, PE, atrial fibrillation, venous thromboembolism, stroke, congestive heart failure, angina pectoris, and genetic or acquired hypercoagulability. There are over 24,000 clinical cases that treated PSS over the years and 77% of cases were reported with cardiovascular, cerebrovascular, hyperlipidemia, and hyperviscositic diseases and the rest with various other cases and were reported to have great efficacy, between 70.3% and 98.3% (Table 4). However, there were only 310 reported adverse cases out of all reported clinical cases (1.29%), including bleeding, edema, leukopenia, allergies, alopecia, anapylatic shock, hypotension, hepatic dysfunctions, muscle pain, priapism, atrioventricular block, and others. Due to the effectiveness of the PSS in clinical application, CFDA subsequently granted 296 drug manufacturers the authority to produce PSS-related drugs where they developed 243 tablets and 53 injection-type drugs. Moreover, PSS has been recommended for combined clinical applications with other approved drugs [166,167].

## 10. Conclusions

Cardiovascular diseases including MI, stroke, arterial thrombosis and venous thromboembolism led by the formation of thrombus are leading causes of mortality throughout the world and are expected to increase over the coming years. The imbalance between the blood coagulation with the natural anticoagulation and their abnormality leads to the formation of a thrombus. Anticoagulants, such as heparin, are widely prescribed in preventing the thrombotic diseases, yet they lead to various complications such as thrombocytopenia and thrombosis syndrome, hemorrhagic complications and platelets activations, which has led to the exploration of effective alternative anticoagulant drugs. Among the various alternatives, from marine organisms derive structurally diverse bioactive substances which can be employed in numerous applications, and they have attracted significant attention toward developing anticoagulants with safety and efficacy. In this review, we discussed the coagulation cascade, presented anticoagulants and their limitations, the structural and potential anticoagulant activity of marine organism-derived macromolecules, and the effect of chemically modified marine organism-derived macromolecules on blood coagulation. In addition, this review provides evidence for further studies to identify and optimize marine-derived substances and chemical modifications to develop promising, safe, and effective anticoagulants.

## Figures and Tables

**Table 1:** Anticoagulant Glycosaminoglycan (from 2010).

Compound	Source	Species	MW	Concentration (μg/mL)	Anticoagulant Activity			Anti-Factor	Additional Findings	Ref
					APTT	PT	TT			
Heparan sulfate	Mollusks	*Nodipecten nodosus*	-	0.001–1	~40–~120 s	-	-	FXa-IC50 0.835 μg/mL fiia- IC50 9.3 μg/mL	In vivo assays demonstrated that at a dose of 1 mg/kg, it inhibited thrombus growth in photochemically injured arteries.	[51]
Heparin/heparan sulfate	Shrimp	*Litopenaeus vannamei*	-	0–15	~40–~250 s	-	-	Inhibit FXa	Anti-Xa activity coupled with low bleeding effects.	[49]
Heparan sulfate	Scallop	*Amussium pleuronectus*	15 kDa	-	135 IU/mg	100 IU/mg	-	-	APTT and PT were lower than standard bovine heparin sulfate.	[59]
Heparan like	Crab	*Goniopsis cruentata*	19 kDa	25–100	~100–~300	-	~175–~300	Inhibit FXa and FIIa	No effect in the extrinsic pathway.	[60]
Heparin/heparan sulfate	Shrimp	*Litopenaeus vannamei*	15 kDa	0.5 μg/mL	-	-	-	Inhibit FIIa	Greater inhibitory effect; 90.7% than heparin.	[61]
LMWH	Shrimp	*Penaeus brasiliensis*	8.5 kDa	5–100	~50->300 s	~15 s	~20–~>300 s	Inhibit FXa and FIIa	Inhibits FXa, HCII.	[62]
Chondroitin sulfate	Smooth hound	*-*	68.78 kDa	25–500	~35–~65 s	~14–~18 s	~20–~60 s	-	Prolong the clotting time APTT, PT, and TT.	[63]
Dermatan sulfate	Pacific starfish	*Lysastrosoma anthosticta*	-	2–10	~30–~100 s	-	-	Inhibit FXa	Prolongs the clotting time.	[64]
Chondroitin sulfate/dermatan sulfate	Corb skin	*Sciaena umbra*	15.46 kDa	25–1000	~30–70 s	~13.5–~19 s	~18–~50	-	Remarkably high anticoagulant, Prolongs the clotting time APTT, PT, and TT.	[58]
Chondroitin sulfate/dermatan sulfate	Corb skin and bone	*Sciaena umbra*	-	25–75	~22–~26 s ~20–~24 s	-	~40–~50 s ~39–~41 s	-	Prolongs the clotting time APTT and TT.	[57]
Fucosylated chondroitin sulfates	Sea cucumbers	*Pearsonothuria graeffei* *Stichopus tremulus* *Holothuria vagabunda* *Isostichopus badionotus*	73–320 kDa 81–340 kDa 100–380 kDa 109–460 kDa	5–65	~30–~50 s ~35–~55 s ~40–~70 s ~45–~75 s		~18–~35 s ~18–~45 s ~25–~50 s ~24–~55 s	-	Prolongs the clotting time APTT and TT., are related to the sulfation pattern.	[65]
Fucosylated chondroitin sulfates	Sea cucumbers	*Stichopus monotuberculatus* *Holothuria scabra* *Apostichopus japonicas* *Holothuria nobilis* *Thelenata ananas*	50–70 kDa 10–15 kDa	-	2.5–7.0 μg/mL	4–23 μg/mL		Inhibit FXa	Stronger AT-dependent anti-FIIa activities and potent HCII-dependent anti-FIIa activities.	[66]
Fucosylated Chondroitin sulfates	Sea cucumbers	*Sostichopus badionotus* *Pearsonothuria graeffei*	-	-	35, 183 U/mg	78,157 μg/mL		Inhibit FXa and FIIa	Prolongs APTT and PT, inhibits FXa, and activates FXII. EC_50_ (Anti-FIIa/HCII); 0.86, 0.05 μg/mL, and EC_50_ Anti-FIIa/ATIII; 12.5, 0.56 μg/mL.	[67]
Fucosylated chondroitin sulfates	Sea cucumbers	*Holothuria scabra*	69 kDa kDa	20–60	~50–~100 s	-	~20–~25 s	-	Prolongs the coagulation and was evaluated by APTT and TT.	[68]
Fucosylated chondroitin sulfates	Sea cucumbers	*Holothuria polii*	45 kDa kDa	5–25	>110s	-	>100	Inhibit FXa and FIIa	High anticoagulant activity mediated by HCII, and to a lesser extent by ATIII.	[69]
Fucosylated chondroitin sulfates	Sea cucumbers	*Cucumaria syracusana*	-	5–25	>100	-	>100	Inhibit FXa and FIIa	High anticoagulant activity mediated by HCII and to a lesser extent by ATIII with IC_50_ of 0.05 μg/mL and 0.09 μg/mL.	[70]
Fucosylated chondroitin sulfates	Sea cucumbers	*-*	3.2–8.8 kDa	-	1.62–8.25 μg/mL	-	-	Inhibit FXa and FIIa	Anticoagulant activities through inhibition of intrinsic tenase, and of FXII.	[71]
Fucosylated chondroitin sulfates	Sea cucumbers	*Isostichopus badionotus*	4.3–109	-	-	-	-	Inhibit FXa and FIIa	High anticoagulant activity mediated by HCII and to a lesser extent by ATIII, results in a significant increase of the anti-FXa /anti-FIIa activity ratio.	[72]
Fucosylated chondroitin sulfates	Sea cucumbers	*Holothuria Mexicana*	-	50–500	~75–>300 s		~20–>800 s	Inhibit FXa and FIIa	Anticoagulant activity is similar to LMWH while inhibiting FIIa and FXa mediated by ATIII.	[73]

**Table 2:** Anticoagulant Glycosaminoglycan mimicking (from 2010).

Compound	Source	Species	MW	Concentration (μg/mL)	Anticoagulant Activity			Anti-Factor	Additional Findings	Ref
					APTT	PT	TT			
Ulvan	Green algae	*Ulva lactuca*	-	0.78–12.50	~29–~74 s	~12–~18 s	~23–>60 s	-	The highest APTT and TT clotting time with high concentrations of acid extracts.	[81]
Ulvan	Green algae	*Ulva lactuca*	185.28 kDa 163.	-	-	-	-	Inhibit FXa and FIIa	Antithrombin-mediated inhibition of FXa and FIIa. The inhibition of venous thrombus formation of rats	[82]
Ulvan	Green macroalga	*Ulva rigida*	-	-	2.5 μg/mL	45 μg/mL	2.62 μg/mL	Inhibit FXa and FIIa	Low ATIII-mediated inhibition activity	[80]
Ulvan	Green algae	*Ulva pertusa*	-	0.6 μg/g 1.2 μg/g 3 μg/g	~62 ~81 ~80	~19 ~25 ~20	-	-	Prolongs the clotting in the male and female Wistar rats	[84]
Low molecular–weight ulvan	Green algae	*Ulothrix flacca*	5 kDa	2.5–50	~48–>200 s	~15–~19	~24–>120 s	-	Mild anticoagulant activities similar to those of LMWH	[83]
Ulvans and their polycarboxyl derivatives	Green algae	*Ulva fasciata*	-	10–150	~28–~220 s	-	-	-	Exhibited a dose-dependent prolongation of APTT	[85]
λ-carrageenan ι-carrageenan	Red algae	*Gigartina skottsbergii*	4.7–3100 kDa 1.1–63 kDa	-	-	-	-	-	High molecular weight λ-carrageenan was comparable to the anticoagulant activity of heparin	[86]
k, k/β, k/ι, λ, iks- carrageenan	Red algae	*Chondrus armatus*, *C. yendoi*, *C. pinnulatus* and *Tichocarpus crinitus*	-	~187, 580, 81, 343, >600, and 59 s	-	-	--		Anticoagulant activity depends on the monosaccharide composition, number, position, and distribution of sulfate	[87]
λ-carrageenan ι-carrageenan	Red algae	Sigma Chemical Co. (St. Louis, MO, EUA)	-	-	240 s 132 s	-	-	-	No anticoagulant action in the PT test	[88]
λ-carrageenan ι-carrageenan θ-carrageenan	Red algae	*Kappaphycus alvarezii* *Eucheuma denticulatum* *Gigartina skottsbergii*	-	10–150	~34–>300 s	-	-	-	Anticoagulant activity depends on molecular weight and/or differences in the sulfation degree or sulfation pattern	[89]
k- carrageenan λ-carrageenan ι-carrageenan ι/υ-carrageenan θ-carrageenan	Red algae	*Kappaphycus alvarezzi* *Gigartina skottsbergii*	36,000 57,800 84,000 70,000 23,700 (g/mol)	-	-	-	-	-	Prolonged coagulation with the carrageenan and oxidized carrageenan	[90]
λ-carrageenan oligosaccharides	Red algae	FMC Biopolymer (Villefranche-Sur-Saône, France)	5.9 kDa	-	-	-	-	Inhibit FXa and FIIa	The anticoagulant activitydepended on the degree of sulfation	[91]
Chitosan-kappa-carrageenan composite hydrogels	Red algae	Aladdin Reagent Co., Ltd.	-	-	110.5 s	-	37.4 s	Attenuate FVIII, IX FIX, XI FXI and FXII	Composite hydrogels had better anticoagulant properties than raw chitosan hydrogels	[92]
Carrageenan-based gel beads	Red algae	Aladdin Reagent Co., Ltd.	100–300 kDa		>600 s	>250 s	~73 s	-	The self-anticoagulant and biocompatible beads prolong the coagulation time significantly	[93]
Fucoidans	Brown algae	*Saccharina japonica*	8.4–50.1 kDa	3.6–14.4	~28–95 s	~8.5–10.5 s	~24–~53 s	-	Prolonged the coagulation dose-dependent manner in APTT ant TT assays	[94]
Heterofucans	Brown algae	*Dictyopteris delicatula*		-	-	-	-	-	No inhibition was in PT and prolonged the APTT	[95]
Xylofucan	Brown algae	*Punctaria plantaginea*	1–5	05–1.4	~4–>100 s	-	-	Inhibit FXa and FIIa	ATIII-medicated anticoagulant activity	[96]
Low molecular fucoidans	Brown algae	*Laminaria japonica*	-	0.7–28	~85–~240 s	~88–~170 s	~53–~160	-	Prolonged the coagulation evaluated by APTT, PT, TT	[97]
Fucan sulfates	Sea cucumber	*Holothuria fuscopunctata* *Thelenota ananas* *Stichopus horrens*	36.8 kDa 61.2 kDa 487.9 kDa	-	11.3 s 10.4 s 19.6 s	-	-	Inhibit FXa and FIIa	Strong inhibition of the intrinsic coagulation pathway through the intrinsic FXase	[98]
Fucan sulfates	Sea cucumber	*Acaudina leucoprocta*	-	2.5–20	~43–~72 s	~9.6–~11.6 s	~13–~13.5s	-	Anticoagulant activity through ATIII activity through HCII.	[99]
Fucan sulfates	Sea cucumber	*Holothuria albiventer*	-	-	~26 μg/mL	-	~116 μg/mL	Inhibit FXa	Prolongation of APTT and TT and intrinsic FXase inhibitory activity	[100]
Fucan sulfates	Sea cucumber	*Pattalus mollis*	6.12–238.3 kDa	-	~20–~23 s	>128	~40–>128s	-	Strong prolongation of coagulation evaluated by APTT and PT	[101]
Rhamnan sulfates	Green algae	*Monostroma angicava*	88.1 kDa	10–100	~33–>200S	~13–~16S	~18–>120	-	Anticoagulant activity mediated by potentiation thrombin by HCII	[102]
Low molecular Rhamnan sulfates	Green algae	Monostroma angicava	24–240 kDa	10–100	~40–~200s	~15–~35	~10–~100	-	Prolongs the clotting time	[103]
Rhamnan sulfates	Green algae	Monostroma angicava	-	5–100	~40–~200 s	~15–~30 s	~10–~120	-	Prolongs the clotting time	[104]
Rhamnan sulfates	Green algae	*Monostroma nitidum*	-	-	-	-	-	Inhibit FXa and FIIa	Anticoagulant activity through inhibition of FXa and FII, inhibits tissue factor expression and von Willebrand factor release	[105]
Low molecular rhamnan sulfates	Green algae	*Monostroma latissimum*.	33.6 kDa	2–50	~30–~200 s	-	-	Inhibit FIIa	Anticoagulant activity mediated by potentiation thrombin by HCII	[106]

**Table 3:** Chemically sulfated polysaccharides and oligosaccharides.

Chemically Sulfated Polysaccharide	Sulfation Technique	MW	Degree of Sulfation/Substitution	Concentration (μg/mL)	In Vitro Anticoagulant Assay			Anti-Factor	Additional Findings	In Vivo or In Silico	Ref
					APTT	PT	TT				
Quaternary ammonium chitosan sulfate	- Quaternary ammonium chitosan by N-(3-chloro-2-hydroxypropyl) trimethyl ammonium chloride - Quaternary ammonium chitosan sulfates by N(SO_3_Na)_3_	-	0.52–1.55 1.55	75 25–75	~90 –~160 s ~90 –~160 s	~10 s ~10–~30 s	~25–~45 s ~40–~50 s	Inhibits FIIa and FXa	Prolonged coagulation Best anticoagulant activity at MW-2.27 × 10^4^.	-	[133]
polyampholytic aryl-sulfonated chitosans	formyl benzene sulfonic acid		<0.803		31–>250 s	11.2–21.8 s	-	Inhibits FXa 0.09 UI/mL	Very low activity on the extrinsic pathway	-	[134]
Silylated chitosan sulfate	sulfur trioxide–pyridine complex in DMSO	18.1–54.5 kDa	1.65–2.46	20–80 s	~-35–~85 s	~13 s	~20–~35 s		Requires a high degree of sulfation (DS > 2.1)	-	[135]
N-propanoyl-, N-hexanoyl- and N,O-quaternary substituted chitosan sulfate	chlorosulfonic acid (ClSO_3_H I) in formamide		0.18–0.81	16.67–66.7s	60.78–138.99 s	0.90–1.11 (INR)	9.60–18.08 s	-	Prolonged coagulation	-	[131]
Carboxybutyrylated hydroxyethyl chitosan sulfate derivative	ClSO_3_H I in N,N-dimethylformamide		0.18–0.77		360 s at 40 μg/mL	-	TT: 20 s at 10 μg/mL	-	Prolonged APTT and TT. Best result when the DS of the carboxyl groups is 0.4/unit	-	[129]
N-succinyl chitosan sulfate	N-succinyl chitosan N(SO_3_Na)_3_	4.5 kDa 13.7 kDa 45.4 kDa 119.6 kDa	1.97	2–75	~86 s ~80 s ~82 s	~25 s ~24 s ~23 s	~20 s ~18 s ~17 s		Depended on DS, MW, and concentration of N-succinyl chitosan sulfate.	-	[130]
Low-molecular-weight chitosan	Oleum to N,N-dimethylformamide	10–50 kDa	1.10–1.63	-	-	-	-	Anti-Xa and anti-IIa activity	Regular increase of anti-Xa activity like heparins	-	[136]
Sulfated chitosan derivative	ClSO_3_H IiH_2_NCHO	-	-	5–100	~25–~175 s	~15–~60 s	~15 s	Inhibit FIIa and FXa	Prolonged coagulation mediated by AT III	-	[127]
Low-molecular-weight chitosan polysulfate	ClSO_3_H I in N,N-dimethylformamide	5.1–26.2 kDa	-	-	40.3–51.7 s	0.88–0.86 (INR)	19.7–12.6 s	Inhibit FIIa and FXa	Prolonged coagulation mediated by AT III and HC II	-	[128]
-N-alkyl derivatives of chitosan sulfate -Quaternary derivatives/chitosan sulfate	ClSO_3_H I in N,N-dimethylformamide		-	-	-	-	-	-	N-alkyl derivatives of chitosan sulfate are more highly potent than Quaternary derivatives/chitosan sulfate	The tail bleeding method in Wistar rats	[126]
Chitosan sulfate (chitosan from *Doryteuthis singhalensis*)	ClSO_3_H I in N,N-dimethylformamide		83.76%	-	6.91 IU/mg	1.85 IU/mg	-	Inhibit FXa	Prolonged coagulation Inhibits FXa through ATIII and thrombin	-	[124]
Chitosan sulfate (chitosan from (*Somanniathelphusa dugasti*)	ClSO_3_H I in N,N-dimethylformamide (obtained 3 fractions of chitosan)		0.21	-	-	21.6–23.2 s	-	Inhibit FXa	Prolonged coagulation Inhibits FXa through ATIII and thrombin	-	[137]
Low molecular weight chitosan sulfate (chitosan from *Sepia pharaonic)*	ClSO_3_H I in N,N-dimethylformamide	1277 Da	-	-	~ 67 s	~ 95 s	-	-	Prolonged the coagulation	-	[138]
Heterochitosans and heterochitooligosacharides	trimethylamine-sulfur trioxide	10–5 kDa 5–1 kDa >1 kDa	-	5–100 5–100 5–100	~37–~44 s ~37–~43 s ~37–~42 s	~15–~25 s ~15–~25 s ~15–~20 s	-	-	Prolonged the coagulation Highest anticoagulant activity: 90% deacetylated chitosan sulfates	-	[139]
Chitosan sulfate (chitosan from *Sepia prashadi*)	ClSO_3_H I in N,N-dimethylformamide	-	-	-	6.90 IU/mg	1.2 IU/mg			Anticoagulant activity depends on sulfate content and the position of sulfate groups.	-	[125]
N-octyl-*O*-sulfate chitosan and derivatives	ClSO_3_H I in N,N-dimethylformamide	150 kDa 400 kDa 600 kDa	-	0–5 0–5 0–5	-	-	-	FXa~100–~10% FXa~100–~6% FXa~100–~6%	Percentage residual activity of factor Xa after inhibition	-	[140]
Chitosan polysulfate (crab shell chitosan from Sigma-Aldrich)	ClSO_3_H I in N,N-dimethylformamide	66 kDa 35 kDa 18 kDa	0.89				27.8 s 22.2 s 22.4 s		Prolonged coagulation Inhibits FXa through ATIII and thrombin	-	[141]
Sulfated alginate	N (SO_3_Na)_3_	-	0.58 0.95 1.25	-	~120–~165 s	~20 s	~38–~42 s		High Degrees of sulfation and concentration inhibit FIIa and FXa Low molecular weight results in higher anti-Xa activity	-	[142]
Sulfonated alginate (Immobilized in membrane_	ClSO_3_H I in N,N-dimethylformamide	-	-	-	>35 s	>14s	>10s	-	Platelet adhesion resistance	-	[143]
Alginate sulfate and quaterized derivatives (QAS-1, QAS-2, and QAS-3)	ClSO_3_H I in N,N-dimethylformamide	-	-	33	~225 s ~200 s ~125 s	~18.5 ~15.5 ~15.5	~22 s ~19 s ~15 s	-	The very high anticoagulant activity of alginate sulfate was reduced by quaternization	-	[144]
Propylene glycol sodium alginate sulfate with low mannuronic acid (M)/guluronic acid (G) ratio	ClSO_3_H I in N,N-dimethylformamide	8403 Da 9446 Da 19716 Da	11.43 11.48 12.27	25	-	~15 s ~15 s ~15 s	~15 s ~20 s ~50 s	Inhibit FIIa	Fractions with low (M)/(G) and high MW prolong APTT and TT, and over-inhibit the FIIa activity mediated by ATIII to induce bleeding risk.	-	[145]
Propylene glycol sodium alginate sulfate with low mannuronic acid (M)/guluronic acid (G) ratio	ClSO_3_H I in N,N-dimethylformamide	8403 Da 9446 Da 19716 Da	11.43 11.48 12.27	25	~50 s ~90 s ~170 s	-	-	-	Prolonged coagulation low M/G ratio or high MW	-	[146]
Propylene glycol sodium alginate and oligosaccharides	-	-	-	5–50	~50–~40 s	~13–~15 s	~10–~40 s	-	Prolonged the APTT, TT, and PT with various fractions, Weaker than heparin	-	[147]
Low-molecular-weight propylene glycol sodium alginate	ClSO_3_H I in N,N-dimethylformamide	~21 kDa ~9 kDa ~7 kDa ~4 kDa ~3 kDa	1.15 1.05 1.01 1.07 1.06	0.78–50	~40–~120 s ~40–~60 s ~40–~80 s ~40–~80 s ~40–~85 s	-	-	Inhibit FIIa	Inhibit FIIa in the presence of ATIII and heparin cofactor II.	Decreased the wet weights and lengths of the thrombus in mice	[148]
Propylene glycol sodium alginate sulfate	ClSO_3_H I in N,N-dimethylformamide	-	-	12.5–200	2.7–>240 s	-	-	-	Prolonged coagulation yet mild anticoagulant	-	[149]
Alginate sulfate (heparin mimetic coating)	H_2_SO_4_ in N,N-dimethylformamide	-	-	1–25	~50–>600 s	-	~0–>24 s	-	Prolonged coagulation and non-coagulation	-	[150]
Alginate sulfate and fragments	ClSO_3_H I in formamide	-	1.75–1.35	75	288 and 102 s	-	-	-	Prolonged coagulation but no increase in PT	-	[151]

**Table 4:** Reported clinical cases and efficacy [166,167].

Disease	Types	Cases	Effective Rate (%)
Hyperviscosity and hyperlipidemia	Hyperviscosity	1518	80.00–96.67
	Hyperlipidemia	3581	75.50–95.08
	Others	81	
Cerebrovascular disease	Ischemic cerebrovascular disease	2666	86.80–98.30
	Cerebral infarction	2689	84.20–95.12
	Stroke prevention and treatment	487	90.00
	Cerebral thrombosis	1294	81.60–96.00
	Others	690	87.04–98.33
Cardiovascular disease	Coronary heart disease	1216	90.00–92.00
	Ischemic heart disease	554	91.30
	Angina	966	77.00–98.08
	Pulmonary heart disease	2156	81.80–97.50
	Others	609	66.70–77.80
